# Correlation between clinical and MRI assessment of depth of invasion in oral tongue squamous cell carcinoma

**DOI:** 10.1186/s40463-016-0172-0

**Published:** 2016-11-22

**Authors:** H. A. Alsaffar, D. P. Goldstein, E. V. King, J. R. de Almeida, D. H. Brown, R. W. Gilbert, P J. Gullane, O. Espin-Garcia, W. Xu, J. C. Irish

**Affiliations:** 1 Department of Otolaryngology - Head and Neck Surgery/Surgical Oncology, Princess Margaret Cancer Centre, University of Toronto, Toronto, ON Canada; 2 Department of Biostatistics, Princess Margaret Cancer Centre, Toronto, ON Canada; 3 Dalla Lana School of Public Health, University of Toronto, Toronto, ON Canada; 4 Department of Otolaryngology - Head and Neck Surgery, University of Ottawa, 501 smyth Rd., K1H 8L6, Ottawa, ON Canada; 5 University of Ottawa, Ottawa, Canada

**Keywords:** MRI, Depth of invasion, Oral tongue SCC, Clinical depth, Pathological depth

## Abstract

**Background:**

Neck metastasis is the most important prognostic factor in oral cavity squamous cell carcinomas (SCC). Apart from the T- stage, depth of invasion has been used as a highly predictable factor for microscopic neck metastasis, despite the controversy on the exact depth cut off point. Depth of invasion can be determined clinically and radio logically. However, there is no standard tool to determine depth of invasion preoperatively. Although MRI is used widely to stage the head and neck disease, its utility in depth evaluation has not formally been assessed.

**Objective:**

- To compare preoperative clinical and radiological depth evaluation in oral tongue SCC using the standard pathological depth.
- To compare clinical and radiological accuracy between superficial (<5 mm) vs. deep invaded tumor (≥5 mm)

**Methods:**

This prospective study used consecutive biopsy-proven oral tongue invasive SCC that presented to the University health network (UHN), Toronto. Clinical examination, radiological scan and appropriate staging were determined preoperatively. Standard pathology reports postoperatively were reviewed to determine the depth of invasion from the tumor specimen.

**Results:**

72 tumour samples were available for analysis and 53 patients were included. For all tumors, both clinical depth (*r* = 0.779; *p* < 0.001) and radiographic depth (*r* =0.907; *p* <0.001) correlated well with pathological depth, with radiographic depth correlating slightly better. Clinical depth also correlated well with radiographic depth (*r* = 0.731; *p* < 0.001). By contrast, for superficial tumors (less than 5 mm on pathological measurement) neither clinical (*r* = 0.333, *p* = 0.34) nor radiographic examination (*r* = − 0.211; *p* = 0.56) correlated with pathological depth of invasion.

**Conclusion:**

This is the first study evaluating the clinical assessment of tumor thickness in comparison to radiographic interpretation in oral cavity cancer. There are strong correlations between pathological, radiological, and clinical measurements in deep tumors (≥5 mm). In superficial tumors (<5 mm), clinical and radiological examination had low correlation with pathological thickness.

## Background

The oral tongue is the most common oral cavity subsite for squamous cell carcinoma (SCC) [1, 2]. Tumor thickness or depth of invasion of 4–5 mm is associated with an increased risk of nodal metastases [3, 4]^.^ The need for elective neck dissection in patients with N0 neck is often based on clinical and/or radiographic assessment of tumor depth of invasion in addition to tumor size. Evaluation of the depth of invasion can be done clinically by tumor palpation and radiographically with the use of magnetic resonance imaging (MRI) [5]. Clinical determination of depth of invasion is limited by one’s ability to determine an exact measurement of depth of invasion based on palpation. Clinical examination may be further hindered by trismus or patient intolerance to palpation due to pain. Assessment of depth of invasion with MRI may be limited by motion artifact due to long image acquisition times and the relative inability to visualize superficial tumors. While there have been studies examining the ability of MRI to predict depth of invasion in oral tongue SCC, these studies have been limited by small sample size, retrospective study design, and exclusion of superficial lesions [6, 7]. Furthermore, none of these studies investigated the value of clinical examination in estimation of depth of invasion. The objective of this study was to correlate preoperative clinical examination and MRI depth of invasion with pathological reported depth of invasion for squamous cell carcinomas of the oral tongue.

## Methods

Research Ethics Board approval was obtained prior to commencement of the study. Patients with a biopsy proven newly diagnosed oral tongue invasive SCC referred to the Princess Margaret Cancer Centre (PMCC) for surgery were prospectively recruited for the study. Patients were excluded if they 1) were referred in with an MRI scan that was reviewed by the surgeon prior to the clinical exam and enrolment in the study 2) had CT imaging only 3) had carcinoma in situ or a previous excisional biopsy or 4) had previous head and neck radiation or chemoradiation. While MRI is the primary modality of choice at the PMCC for staging oral tongue cancers, CT scans are used in cases where patients come with a CT which is deemed adequate to stage the tumor or have contraindications to an MRI. For both MRI and pathologic examination, tumor depth of invasion was measured from the adjacent normal mucosa to the deepest aspect of tumor. The treating head and neck surgeon estimated clinical depth of invasion based on palpation prior to radiographic evaluation of tumor depth. Two head and neck radiologists reviewed the MRIs independently and were blinded to the clinical depth. An average of the two readings was used for analysis. All patients underwent surgical resection with or without a neck dissection. Depth of invasion was assessed on gross pathologic examination and histopathologic examination on formalin fixed specimen.

Statistical analyses were performed using Version 9.2 of the SAS (SAS Institute, Cary, NC). Correlation between clinical examination, radiographic examination, and pathological depth was performed using Spearman correlation. Agreement between the three depths of invasion was measured using the Kappa coefficient. Two-sided tests were applied. Results were considered significant if the p-value was less than or equal to 0.05.

## Results

Fifty-three consecutive patients that met the criteria for inclusion were enrolled in the study between December 2009 to December 2011. All patients had surgical resection of the primary tumor with a partial glossectomy, 40 of which also had a selective neck dissection upon the surgeon discretion. Patient demographics are presented in Table 1. The correlation between the two radiologists measurement of depth of invasion on MRI was 0.64 with (95 % C.I. 0.43 - 0.84, *p* <0.001). The mean depth of invasion determined radiologically, clinically, and pathologically were 10.9 mm, 10.2 mm and 11.2 mm, respectively (Table 2). On pathologic examination 10 patients had superficial tumours (<5 mm) and 43 had deep tumours (≥5 mm).

**Table 1:** Patient demographics and preoperative TN staging

Gender	
Male	34
Female	19
Mean age	64
T stage	
T1	22
T2	22
T3	7
T4	2
N stage	
N0	32
N1	7
N2	11
N3	0

**Table 2:** 53 Correlation between MRI, clinical and pathological depth of invasion measurements

Measures	Obscured	N	Mean	St.Dev
MRI depth	4	49	10.9	6.6
Clinical depth	0	53	10.2	8.4
Pathologic depth	0	53	11.1	7.9

Depth measurements were obtained. 4 radiological measures were obscured by both radiologists

The correlation between clinical and pathologic depth of invasion was significant (*r* = 0.78; *p* < 0.001) as was the correlation between depth of invasion reported on MRI and pathologic depth of invasion (*r* =0.91; *p* <0.001). The correlation between depth of invasion on clinical examination and MRI was also significant (*r* = 0.731; *p* < 0.001). For tumors less than 5 mm on pathological measurement, neither clinical (*r* = 0.333, *p* = 0.34) nor radiographic examination (*r* = − 0.211; *p* = 0.56) correlated well with pathological depth. Additionally, in this superficial tumour cohort, clinical and radiographic depth of invasion did not correlate well with each other (*r* = −0.064; *p* = 0.86). In contrast, for tumors measuring ≥ 5 mm in depth both clinical (*r* = 0.757; *p* <0.001) and radiographic measurement (*r* = 0.856; *p* < 0.001) of depth of invasion correlated well with pathological depth of invasion. For these thicker tumors the correlation between clinical and radiographic depth of invasion was *r* = 0.645 (*p* <0.001).

For the entire cohort, the sensitivity and specificity of clinical examination in determining the depth of invasion (<5 mm or ≥ 5 mm) with pathology as the gold standard was 80 % and 84 %, respectively (Table 3). The sensitivity and specificity of MRI assessing the depth of invasion < 5 mm or ≥ 5 mm was 80 % and 97 %, respectively (Table 4).

**Table 3:** Sensitivity and specificity of clinical depth in comparison to pathological depth

Clinical depth (mm)		Pathological depth	
	<5	≥5	Total
<5	8	5	13
≥5	2	38	40
Total	10	43	53
Sensitivity		80 %	
Specificity		84 %	
PPV		61.5 %	
Kappa coefficient		0.613, 95 % C.I (0.36-0.87)	

Kappa coefficients were used to determine the agreement between measures once categorized according to the cutoff point. Closer values to 1 mean higher agreement between categories

**Table 4:** Sensitivity and specificity of radiological depth in comparison to pathological depth

Rad. Depth (mm)	Pathological depth		
	<5	≥5	Total
<5	8	1	9
≥5	2	38	40
Total	10	43	49
Sensitivity	80 %		
Specificity	97 %		
PPV	89 %		
Kappa coefficient	0.804, 95 % C.I (0.59-1.00)		

Kappa coefficients were used to determine the agreement between measures once categorized according to the cutoff point. Closer values to 1 mean higher agreement between categories

## Discussion

Occult metastasis to the cervical lymph nodes may occur in up to 40 % of patients with early stage (T1and T2) tongue cancers [4]. One of the primary predictors of nodal metastases and determinants of prognosis in these patients is tumor depth of invasion [8]. Therefore, it is important for clinicians to be able to determine the extent of tumor depth of invasion preoperatively. This would help to decide on elective neck dissection, the extent of surgery, reconstruction, and provide patients with an idea as to whether they may require post-operative radiation. Herein we report on utility of clinical examination and MRI in determining depth of invasion of oral tongue SCC.

In this study, clinical examination and MRI were both adequate at determining depth of invasion compared with final pathology when tumors were ≥ 5 mm in depth, but not for those less than 5 mm. We used 5 mm as a cutoff as this is the depth at which the risk of nodal metastases increases, based on the literature [3, 4]. Since the clinical importance is to be able to detect deeper tumors, the decreased ability of either examination to be able to accurately predict the depth of superficial lesions is less clinically significant.

There have been previous studies investigating the accuracy of MRI in predicting the depth of invasion of oral tongue SCC, however these studies primarily have a small sample size and retrospective study design, and none have compared MRI with clinical examination. Preda et al. investigated 33 oral tongue SCC in a retrospective series [9]. The authors demonstrated that MRI thicknesses correlated strongly with histological tumor thicknesses (correlation coefficient = 0.68, *p* < 0.0001). Park et al., evaluated 114 patients with oral cavity and oropharyngeal SCC of which 49 patients had oral tongue SCC. Relationship between MRI and histologic depth of invasion in oral tongue subsite was high with a correlation coefficient of 0.949 [7]. In this study, the mean depth of invasion by histology and MRI were 13.57 mm, 15.24 mm respectively. This group reported on deeper tumours, explaining the better correlation.

As pointed out by Lawein et al., there is tumor shrinkage after resection affecting all oral cavity subsites, including the oral tongue. The tumor shrinkage factor for oral tongue cancer has been reported to be 87 % [6]. Most of the studies assessing the relationship between tumor depth of invasion and risk of nodal metastases are based on pathologic assessment and not clinical or radiographic assessment. Therefore, clinical and MRI examination may under or over estimate depth of invasion and may not have the same ability to predict nodal metastases.

Sentinel lymph node (SLN) biopsy has been evaluated in the recent years in head and neck cancer. A few studies that evaluated SLN for oral and oropharyngeal cancer however most of these studies included advanced T stage and did not study specific subsite [12–14]. Sagheb et al. did a pilot study to examine the role of SLN in early T stage tongue SCC with N0 neck. A SLN was followed by a neck dissection during the same operation [15]. It was concluded that the sensitivity of SLN is about 75 % and further investigation is needed.

While MRI was shown to correlate well with pathological depth and is more sensitive and specific for depth measurements than clinical assessment, the latter test is complementary and useful in situations where either MRI is unavailable or difficult to interpret due to artefacts. In a prospective study, Yeun et al. examined the correlation between ultrasound and pathologic tumor thickness in 45 oral tongue carcinoma patients during general anaesthesia and before commencing surgery [10]. There was a statistically significant correlation coefficient of 0.940 (*p* < .005). While this technique may be difficult to perform in clinic due to pain or trismus, its improved ability to measure tumor thickness does warrant further investigation.

Despite the importance of depth of invasion, other histopathological parameters have been found to correlate with nodal metastasis including size of the tumor in greatest dimension, and other pathologic features such as pattern of invasion, density of cancer-associated fibroblasts, perineural, and vascular invasion [11]. All these need to be taken in account to determine the risk of regional metastasis.

As far as we are aware, this is the first study to evaluate the clinical assessment of tumor thickness in comparison to radiographic interpretation. There are strong correlations between pathological, radiological, and clinical measurements. We have shown that for deep tumors, clinical palpation is an acceptable assessment modality to determine thickness of oral tongue carcinoma. It is an acceptable adjunct to MRI to assist pre-treatment staging and prognosis evaluation. Additional techniques are needed to better assess superficial lesions and these may include US.

## Conclusions

This is the first study evaluating the clinical assessment of tumor thickness in comparison to radiographic interpretation in oral cavity cancer. There are strong correlations between pathological, radiological, and clinical measurements in deep tumors (≥5 mm). In superficial tumors (<5 mm), clinical and radiological examination had low correlation with pathological thickness.

## Abbreviations

MRI: Magnetic resonance imaging
SCC: Squamous cell carcinomas
SLN: Sentinel lymph node biopsy
